# Clinical factors, C-reactive protein point of care test and chest X-ray in patients with pneumonia: A survey in primary care

**DOI:** 10.1080/13814788.2019.1649651

**Published:** 2019-08-28

**Authors:** Geert H. Groeneveld, Robert J. van de Peppel, Margot W. M. de Waal, Theo J. M. Verheij, Jaap T. van Dissel

**Affiliations:** a Department of Internal Medicine, Leiden University Medical Center, Leiden, The Netherlands;; b Department of Infectious Diseases, Leiden University Medical Center, Leiden, The Netherlands;; c Department of Public Health and Primary Care, Leiden University Medical Center, Leiden, The Netherlands;; d Julius Center for Health Sciences and Primary Care, University Medical Center Utrecht, Utrecht, The Netherlands;; e Centre for Infectious Disease Control, Dutch National Institute for Public Health and the Environment, Bilthoven, The Netherlands

**Keywords:** C-reactive protein, general practice, pneumonia, respiratory tract infections, X-rays

## Abstract

**Background:** In patients with an acute lower respiratory tract infection (LRTI), general practitioners (GPs) often find it challenging to decide to prescribe antibiotics or not. C-reactive protein (CRP) point of care test (POCT), and chest X-ray are diagnostic tests that can optimize the treatment decision. However, their usefulness in clinical practice is unknown.

**Objectives:** To determine the proportion of Dutch GPs using CRP and chest X-ray in patients with an acute LRTI. To determine whether clinical factors and C-reactive protein point of care test affect the behaviour in requesting chest X-rays.

**Methods:** In 2014, a questionnaire was sent to a random sample of 900 Dutch GPs. Outcome parameters are the use of CRP and chest X-ray, the percentage of GPs who guide their decision in requesting chest X-rays by CRP testing and the GP’s expectation regarding presence or absence of pneumonia. In addition, considerations for requesting chest X-rays were assessed.

**Results:** Two hundred and fifty-five completed questionnaires (29%) were returned. In 2014, 54% of the responding GPs used the CRP test. These GPs tend to use fewer chest X-rays (*p* = 0.07). GPs overestimate the chance that pneumonia will be present on the radiograph. Seventy percent consider the possibility of abnormalities other than pneumonia as the main reason for requesting a chest X-ray.

**Conclusion:** In patients with an acute lower respiratory tract infection, GPs report that CRP results affect their behaviour regarding the request of a chest X-ray in patients with lower respiratory tract infection and therefore research is needed to substantiate the use of these diagnostic tools for this purpose.

## Introduction

KEY MESSAGES

- A vast majority of GPs use the CRP test and most of them requested a chest X-ray on CRP result.
- GPs that use CRP test reported to request fewer chest X-rays in patients with an acute lower respiratory tract infection.
- GPs overestimate the chance of finding pneumonia on chest X-rays.

In patients that present with an acute lower respiratory tract infection (LRTI), the decision whether or not to prescribe antibiotics is sometimes complex, especially in moderately ill patients [1,2]. Dutch general practitioners (GPs) use antibiotics more restrictively than their colleagues in other European countries [3]. However, there are also substantial regional differences within the Netherlands [4]. These differences are an expression of the complexity of the consideration of whether or not to prescribe an antibiotic. In general, one can state that patients with acute bronchitis do not need antimicrobial treatment while patients with pneumonia do [5,6]. Unfortunately, for the diagnosis of pneumonia, the use of anamnesis and physical examination alone provide insufficient support [7–9].

Two types of additional (diagnostic) tests for acute LRTI can be used in general practice: the C-reactive protein point of care test (CRP POCT) and the chest X-ray. A low CRP (<20 mg/l) can exclude pneumonia with reasonable certainty, irrespective of clinical findings while an elevated CRP (>100 mg/l) greatly increases the chance of pneumonia warranting antibiotic treatment [8,10]. A recent meta-analysis ascertained that even when clinical variables are taken into account, the CRP test can help to confirm or exclude pneumonia [11]. Different guidelines (e.g. the British and the Dutch guidelines) therefore, indicated the use of the CRP test in moderately ill patients [1,12]. Studies that evaluated whether the CRP POCT reduced the number of antibiotic prescriptions showed variable results [13,14].

A chest X-ray can be used to detect pneumonia but the use of this examination in all individuals in whom pneumonia is suspected is not recommended. The chest X-ray is currently only recommended in the Dutch guideline to investigate, the cause of lack of recovery, uncertainty about the diagnosis or treatment, or when a condition other than pneumonia is suspected as an explanation for the symptoms [1]. The British guideline does not mention the chest X-ray as a diagnostic tool in patients with suspected pneumonia or exacerbations of asthma and COPD. Every year, GPs request about 31 chest radiographs per 1000 person-years [15]. Research into the effectiveness of requesting chest X-rays by the GP for certain subgroups of patients with an acute LRTI is lacking. The objective of this study was to assess the use of chest X-ray and the CRP POCT in patients with an acute respiratory tract infection in Dutch primary care. We asked the GPs about their estimates and experiences with this complex situation where evidence for a specific strategy is lacking.

## Methods

### Study design and setting

Between May and September 2014, a questionnaire-based cross-sectional study was performed in the Netherlands. The registry from the Netherlands Institute for Healthcare Research (NIVEL) contains address information of all GPs in the Netherlands. A random sample of 900 addresses was drawn. The questionnaire (Supplementary Appendix) was sent in May 2014 per mail to these family practice addresses.

### Construction of the questionnaire

The two principal investigators (GHG and RJP) held an exploratory focus group discussion with various GPs in the Leiden region, the Netherlands. In this discussion, open questions were asked about the way in which the GPs use additional diagnostic tests in patients with acute LRTI and in what way the results of the tests affect their treatment policy [16]. An acute LRTI was defined as complaints for less than three weeks.

Based on the results, a list with open and closed questions was generated and distributed among 15 GPs in the Leiden region via the newsletter of the Leiden Primary Care Research Network. The answers and feedback received via this route contributed to the final quantitative questionnaire.

### Quantitative questionnaire

First, the questionnaire asks about the number of years of work experience, the number of hours a week that the GP works at the general practice, and an estimate of the number of chest X-rays requested in a year for patients with acute LRTI.

Main outcomes are the use of CRP POCT, the percentage of GPs who guide their decision in requesting chest X-rays by CRP testing and the expectation regarding presence of pneumonia on chest X-rays. In addition, indications for the use of CRP POCT, clinical parameters and distribution of reasons for requesting chest X-rays (in GPs with and without CRP test available), which other pathology the GP wants to exclude and diagnostic and therapeutic consequences when pneumonia is present or absent were assessed.

Various characteristics and consequences could be scored on five-point Likert scales, with answers varying from ‘(almost) never’/‘very unimportant’ to ‘(almost) always’/‘very important.’ The complete questionnaire is available in the Supplementary Appendix.

### Analysis

The returned questionnaires were anonymized. Descriptive analyses and comparison of proportion with chi-squared test were performed with SPSS (IBM SPSS Statistics for Windows, Version 23.0., Armonk, NY).

## Results

### Study population

Twenty-three questionnaires were returned due to outdated address details. In total, after one reminder letter, 255 of the 877 (29%) questionnaires were returned completed in September 2014. The respondents reported a median work experience of 14 years, (interquartile range, IQR, 9–22 years) and a median workweek of 36 h (IQR: 30–41.5 h) at the general practice.

### Chest X-ray

Median reported the number of chest X-rays per year for patients with an acute LRTI was 10 (IQR: 4–12). The 24 respondents (9%) that never requested a chest X-ray for this indication could not answer the remaining questions. Median work experience and work week in the respondents who never request a chest X-ray did not differ from respondents who did request chest X-rays.

Tables 1 and 2 provide an overview of the reports of GPs regarding considerations and objectives to request a chest X-ray. Most GPs (70%) consider the detection or exclusion of abnormalities other than pneumonia as one of the main reasons for requesting a chest X-ray. The exclusion of malignancy, heart failure, sarcoidosis, and tuberculosis are mentioned repeatedly. If the chest X-ray has been required to exclude other pathology, the GP will state this in 90% of the cases on the X-ray application form. Factors that play an essential role in the decision to request a chest X-ray are mainly age, smoking, and the duration of the complaints.

**Table 1.** Questionnaire response from general practitioners: Clinical factors in the consideration to request a chest X-ray in patients with an acute lower respiratory tract infection (*n* = 226^a^).

Clinical factors in the consideration to request a chest X-ray	Rating	
	Important (%)	Neutral or unimportant (%)
Smoking	191 (85)	35 (15)
Duration of the complaints	186 (82)	40 (18)
Age	179 (79)	47 (21)
Presence of fever	98 (43)	128 (57)
Duration of fever	95 (42)	131 (58)
Response to previous antibiotics	92 (41)	134 (59)
Producing sputum, and sputum colour	28 (12)	198 (88)

a 29 respondents never requested chest X-rays and/or did not give an answer to this question.

**Table 2.** Questionnaire response from general practitioners: Reasons to request a chest X-ray in patients with an acute lower respiratory tract infection (*n* = 228^a^).

Reasons to request a chest X-ray	Number of times indicated to be the most important (%^b^)
Detection or exclusion of other lung abnormalities, such as a lung tumour	159 (69.7)
Confirm the diagnosis of pneumonia	87 (38.2)
Exclude the diagnosis of pneumonia	76 (33.3)
Reassuring the patient	22 (9.6)
Uncertainty about further policy	21 (9.2)
As a guide to decide on antibiotic … prescription	18 (7.9)
Conditions that GPs want to exclude	Number of times indicated (%), *n* = 190^c^
Lung cancer	160 (84.2)
Heart failure	46 (24.2)
Sarcoidosis	36 (18.9)
Tuberculosis	24 (12.6)
Pneumothorax	15 (6.9)
Other^d^	48 (25.2)

a 27 respondents never requested chest X-rays and/or did not give an answer to this question.

b Percentages add up to > 100% because some respondents gave more than one reason the same score.

c Some GPs who did not state the exclusion of other lung abnormalities as the most important reason also answered this question; in addition, several answers could be filled in.

d Other disorders included foreign body, pulmonary embolism, and systemic lupus erythematosus and were each mentioned by <5% of all respondents.

**Figure 1.**
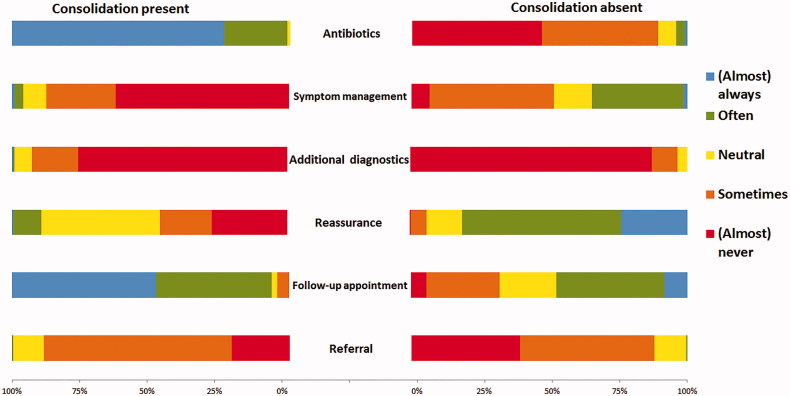
Questionnaire response from general practitioners: Policy following the chest X-ray in patients with an acute lower respiratory tract infection (*n* = 230^a^). Bi-directional bar chart. On the left the policy followed in case a pneumonia was detected on the chest X-ray, on the right the policy followed in case no pneumonia was detected on the chest X-ray. In the middle, description of the policy. ^a^24 respondents never request a chest X-ray and one did not answer this question.

The assumption of 217 GPs (14 GPs did not answer this question and 24 never requested a chest X-ray) to detect a lung infiltrate on the chest X-ray was less than 10% in 13% of GPs, between 10 and 20% in 19% of GPs, and more than 50% in 68% of GPs. If an infiltrate suspect for pneumonia is present, 227 of the 230 GPs (99%; 1 GP did not answer this question and 24 GPs never request a chest X-ray) often, to almost always, prescribe an antibiotic. In the absence of pneumonia, 4% of GPs often, to nearly always, prescribe an antibiotic (Figure 1).

### CRP point of care test

The CRP POCT is used by the vast majority of GPs (54%). Most GPs also use the test to evaluate suspected infections other than pneumonia (Table 3), e.g. diverticulitis, urinary tract infection, or an unknown ‘other’ infection. GPs (80%) reported that they foresee that CRP POCT can replace chest X-ray as a diagnostic test partially or completely. GPs with CRP test available are more confident than those that do not have this test available (86% vs 71%, *p* <0.01).

**Table 3.** Questionnaire response from general practitioners: Use and indications for use of the CRP point of care test (*n* = 246^a^).

	Number (%)
Respondents that use the CRP point of care test in the general practice	134 (54)
Use only if there is a suspicion of respiratory tract infection	35 (26)
Use in case of suspected respiratory tract or other infection	83 (62)
Hardly ever use the test	16 (12)
In many cases, the CRP point of care test plays a role in the consideration to request a chest X-ray^b^	75 (56)
Respondents that do not use the CRP point of care test in the general practice	112 (46)
Would like to purchase the test in the future	85 (76)
Would not like to purchase the test in the future	27 (24)

a Nine GPs did not answer this question.

b Respondents that indicated that this ‘often’ or ‘(almost) always’ plays a role.

### Difference between GPs with and without CRP test

GPs with CRP POCT available reported requesting fewer chest X-rays than their colleagues without CRP POCT available (median 6, IQR: 3–10 vs median 10, IQR: 5–14, respectively; *p* = 0.07).

Expectation regarding the presence of pneumonia did not differ between GPs with or without CRP POCT available (*p* = 0.67).

Presence and colour of sputum were reported to be more critical when considering chest X-ray by GPs without, than those with, CRP POCT available (Figure S1).

Guidance whether or not to prescribe antibiotics is reported as a reason for requesting chest X-ray less frequently in GPs with CRP than in GPs without CRP. Other reasons were not different (Figure S2).

GPs who do not use CRP POCT reported more frequently than those who do use CRP POCT starting symptom management in case pneumonia is confirmed (neutral to almost always 15% vs 9%; *p* = 0.05) or ruled out with chest X-ray (neutral to almost always 57% vs 41%; *p* <0.01). The remaining policy items did not differ significantly between GP groups.

## Discussion

### Main findings

This study shows that in 255 Dutch GPs, the use of additional diagnostic tools for the suspicion of acute LRTI was diverse. GPs reported estimating the probability of having pneumonia as high among patients for whom they request a chest X-ray. Nearly 70% of GPs request the photo mainly to exclude other pathology. The vast majority of GPs had the CRP POCT available in 2014 and most GPs used this test to determine whether or not to request a chest X-ray. GPs using CRP POCTs reported requesting fewer chest X-rays than GPs who did not use this test. These latter GPs reported using chest X-ray more often to guide the decision to prescribe antibiotics. Many GPs also used the CRP POCT for other purposes.

### Strengths and limitations

The strengths of this study are the random sample of GPs in the Netherlands and the considerable number of 255 completed surveys that were available for analysis. The inventory based on focus group interview and pilot questionnaires during the pilot study means that the diversity of ideas, experiences, and behaviours in the target group were well explored. The fact that both GPs with and without a CRP POCT, as well as GPs that vary from never to frequently requesting chest X-rays have responded, means that the sample has included all extremes of diagnostic policy.

A limitation of the study is the potential occurrence of sampling bias. The ‘selection’ of respondents could be different from that of the GPs who did not respond. Although the absolute number of questionnaires analysed is considerable, the response rate of 29% is not high. A review by Creavin et al. showed a mean response rate of 61% [17]. However, the response rate in recent surveys among Dutch GPs is substantially lower [17–19]. Respondents could be more interested in this topic than non-responders and thereby more aware of guidelines and evidence, resulting in more prudent use of diagnostic tools. The years of work experience and the number of working hours of the respondents correspond to the national average, 14.9 years and 31.2 h per week, respectively [20]. Moreover, McFarlane et al. demonstrated that higher response rates in a survey of physicians are not associated with lower selection bias [21].

Nonetheless, the potential difference in characteristics between GPs who filled in the questionnaire and the ones that did not respond might still be present. However, the study provides a useful insight into the considerations of the Dutch GP about additional diagnostic tools for acute LRTIs.

Not every possible consideration has been asked in this short questionnaire. For example, it is not clear in what type of patient the CRP POCT is used, if CRP kinetics are taken into consideration, and how GPs interpret the results. A previous study showed that most GPs use the CRP POCT in patients who are moderately ill when it is not immediately obvious whether or not the patient needs an antibiotic. In the same study, it was found that the CRP POCT is sometimes used too frequently, even in situations where this test should have no consequences for the policy [22].

This is a survey-based study about opinions and perceptions, which do not necessarily reflect the real management and prescription habits. The survey was completed in 2014. It is possible that with an increase in use, the interpretation of the results will also change slightly.

### Interpretation

GPs’ expectation about the likelihood of detecting a lung infiltrate on the X-ray is high. Two-thirds expect an infiltrate in more than 20% of the patients. This estimate does not match the findings in several primary care studies, where pneumonia on the chest X-ray was detected in only 5–13% [8,11,23].

The chest X-ray is the gold standard for the detection or exclusion of pneumonia, while clinical features, including a low CRP value, can safely exclude pneumonia [11,12]. The benefit of chest X-rays in the detection or exclusion of pneumonia is, therefore, primarily present in the group of patients with a high probability of the presence of an infiltrate. This mainly concerns patients with clinical characteristics fitting with pneumonia that have an elevated CRP value. We hypothesize that GPs may request too many chest X-rays because they overestimate the likelihood of pneumonia. With better pretest (pre-chest X-ray) assessment, for example by using CRP, pneumonia could be ruled out more often without chest X-ray. Differently, GPs incorrectly withhold some patients from chest X-rays because they do not adequately determine the group of patients with a high pretest (pre-chest X-ray) probability, partially because only 54% in our study used the CRP test. Additionally, given the discrepancy between the pretest assessment and the actual percentage of pneumonia present on lung images, pneumonia can often be excluded with a chest X-ray. In the latter case, antibiotics are prescribed less frequently.

The lack of evidence is the reason that the chest X-ray is currently not clearly defined in the standard of the Dutch Society of GPs or the British guidelines for the detection or exclusion of pneumonia [1,12]. However, this study shows that GPs already use the results of the CRP test in their decision to request a chest X-ray and/or they foresee that the CRP test can replace the chest X-ray as a diagnostic tool.

Often the detection or exclusion of a condition other than pneumonia is indicated as the main reason to request a chest X-ray. In a European cohort of nearly 3000 patients with an acute cough who underwent a chest X-ray, a clinically relevant abnormality—other than pneumonia—was found in 3% [24]. Therefore, the chance that a GP will find such aberrations is small. Malignancy can be missed on the chest X-ray, especially if at that time an infiltrate is present in the same area. It is then preferable to repeat the chest X-ray after pneumonia has been treated [25].

Accurate information about the availability and use of CRP POCT in European countries is not known. Oppong et al. reported that CRP POCT was available in 12 of 14 primary care networks in 13 European countries [26]. There were marked differences in the availability of CRP tests between Spain and Denmark and between CRP use in Belgium (3%) [27], the UK (15%), and the Netherlands (48%) in 2012–2013 [28]. The use of CRP has increased in Scandinavian countries between 2004 and 2013 [29].

When comparing Danish primary care versus Spanish primary care, chest X-rays are used more frequently to confirm pneumonia in Spain [27].

### Implications

With the frequent use of the CRP POCT to aid in the decision to request a chest radiograph, there appears to be a need for research into a diagnostic algorithm, that would incorporate clinical characteristics and a CRP result, to determine in which patient a chest X-ray has added value.

This study also shows that GPs using the CRP POCT frequently use it for infections other than pneumonia. The use of the CRP test is only recommended for patients with acute LRTIs or diverticulitis. For both disorders, the use of the CRP test has many limitations [1,30]. Restraint in its use is therefore required until new research proves that either the CRP POCT has added value for other indications, or that the CRP test can replace a chest radiograph.

## Conclusion

GPs widely use the CRP POCT and often base their decision to request a chest X-ray on the outcome. They overestimate the chance of finding pneumonia in these patients. Clinical variables in combination with the CRP POCT could help the GP to request chest radiographs more selectively for patients with acute LRTI. Research, however, is first needed to substantiate the use of these diagnostic tools for this purpose.

## Supplementary Material

Original questionnaire

Figure S2. Bidirectional bar chart questionnaire response from general practitioners about reasons to request a chest X ray in patients with an acute RTI

Figure S1. Considerations to request a chest X ray with or without CRP
