# Supplementary material for: Clinical factors, C-reactive protein point of care test and chest X-ray in patients with pneumonia: A survey in primary care
Source: Eur J Gen Pract. 2019 Aug 28;25(4):229–35. doi: 10.1080/13814788.2019.1649651 (PMC6853238; doi:10.1080/13814788.2019.1649651)
Supplement: Original questionnaire [file IGEN_A_1649651_SM6412.pdf]

LEIDS UNIVERSITAIR MEDISCH CENTRUM

Een thoraxfoto bij verdenking op een acute luchtweginfectie in de huisartspraktijk

In Nederland worden ieder jaar naar schatting 500.000 thoraxfoto's aangevraagd door de huisarts, waarvan ongeveer 25% op verdenking van een acute luchtweginfectie. In uw praktijk komen jaarlijks waarschijnlijk vele patiënten op het spreekuur met een klachtenpatroon dat bij een acute luchtweginfectie past. Op dit moment doet de afdeling Infectieziekten van het LUMC onderzoek naar de waarde van de thoraxfoto bij het beleid bij lagere luchtweginfecties in de regio Leiden en Den Haag.

Deze enquête richt zich op de vraag hoe u de thoraxfoto als diagnosticum inzet bij patiënten die zich presenteren met klachten die kunnen passen bij een acute luchtweginfectie. Tot slot willen wij een paar korte vragen stellen over het beleid na de uitslag van de foto en het eventuele gebruik van een CRP-bepaling.

1. Hoeveel jaar werkt u al als huisarts?

☐ Jaar

2. Hoeveel uren per week werkt u op uw praktijk?

☐ uur per week

3. Hoeveel thoraxfoto's vraagt u ongeveer per jaar aan voor patiënten die mogelijk een acute luchtweginfectie hebben (< 3 weken klachten)?

☐ per jaar (*Indien u voor deze indicatie nooit een thoraxfoto aanvraagt, gelieve hier "0" in te vullen en verder te gaan naar vraag 11*)

4. Wat is voor u de belangrijkste reden om een thoraxfoto aan te vragen bij deze patiënten?

	**(Bijna) nooit**	**Soms**	**Neutraal**	**Vaak**	**(Bijna) altijd**
Bevestigen van de diagnose van een lagere luchtweginfectie/pneumonie	○	○	○	○	○
Uitsluiten van de diagnose van een lagere luchtweginfectie/pneumonie	○	○	○	○	○
Als houvast om wel of geen antibiotica voor te schrijven	○	○	○	○	○
Aantonen of uitsluiten van andere longafwijkingen, zoals een longtumor	○	○	○	○	○
Geruststellen van de patiënt, ook al hebt u geen (medische) indicatie de foto aan te vragen	○	○	○	○	○
Omdat u onzeker bent over het verdere beleid	○	○	○	○	○
Anders, namelijk:	○	○	○	○	○

5. Als u een patiënt voor deze indicatie voor een thoraxfoto stuurt, wat is dan uw verwachting met betrekking tot het aantonen van een longinfiltraat?

- ◯ 0% - 5%
- ◯ 5% - 10%
- ◯ 10% - 20%
- ◯ 20% - 50%
- ◯ > 50%

5. Welke van deze factoren vindt u belangrijk in de afweging om voor een dergelijke patiënt een thoraxfoto aan te vragen?

	**Zeer onbelangrijk**	**Onbelangrijk**	**Neutraal**	**Belangrijk**	**Zeer belangrijk**
De leeftijd van de patiënt	◯	◯	◯	◯	◯
Of de patient rookt	◯	◯	◯	◯	◯
Het opgeven van sputum en de kleur van het sputum	◯	◯	◯	◯	◯
De reactie op voorgaande antibiotica	◯	◯	◯	◯	◯
De duur van de klachten	◯	◯	◯	◯	◯
De aanwezigheid van koorts	◯	◯	◯	◯	◯
De duur van de koorts	◯	◯	◯	◯	◯

7. Indien u wel eens een thoraxfoto bij deze patiënten aanvraagt om andere pathologie uit te sluiten, welke andere pathologie bedoelt u dan?

8. Als u een thoraxfoto aanvraagt bij deze patiënten om andere pathologie uit te sluiten, schrijft u dat dan ook op het aanvraagformulier?

- ◯ (Bijna) Nooit
- ◯ Soms
- ◯ Neutraal
- ◯ Vaak
- ◯ (Bijna) altijd

9. *U heeft een thoraxfoto aangevraagd bij een patiënt die u verdenkt van een acute luchtweginfectie (< 3 weken klachten). De foto toont **geen afwijkingen**.*

Welke consequenties trekt u hieruit?

	**(Bijna) nooit**	**Soms**	**Neutraal**	**Vaak**	**(Bijna) altijd**
U schrijft toch een antibioticum voor	◯	◯	◯	◯	◯
U besluit klachten te bestrijden, bijvoorbeeld met codeïne	◯	◯	◯	◯	◯
U besluit tot aanvullend onderzoek, bijvoorbeeld een CT-scan	◯	◯	◯	◯	◯
U stelt de patiënt gerust	◯	◯	◯	◯	◯
U maakt een vervolgafspraak	◯	◯	◯	◯	◯

U verwijst de patiënt door	◯	◯	◯	◯	◯

10. *U heeft een thoraxfoto aangevraagd bij een patiënt die u verdenkt van een acute luchtweginfectie (<3 weken klachten). De foto toont* ***een longinfiltraat, verdacht voor pneumonie****.*

Welke consequenties trekt u hieruit?

	(Bijna) nooit	Soms	Neutraal	Vaak	(Bijna) altijd
U schrijft een antibioticum voor	◯	◯	◯	◯	◯
U besluit klachten te bestrijden, bijvoorbeeld met codeïne	◯	◯	◯	◯	◯
U besluit tot aanvullend onderzoek, bijvoorbeeld een CT-scan	◯	◯	◯	◯	◯
U stelt de patiënt gerust	◯	◯	◯	◯	◯
U maakt een vervolgafspraak	◯	◯	◯	◯	◯
U verwijst de patiënt door	◯	◯	◯	◯	◯

11. Gebruikt u de CRP-sneltest in uw praktijk?

- ○ Ja, bij een vermoeden op een luchtweginfectie
- ○ Ja, bij een vermoeden op een luchtweginfectie of een overige infectie
- ○ Ja, maar ik gebruik de test (bijna) nooit
- ○ Nee, maar ik zou de test wel willen gebruiken
- ○ Nee, en ik zou de test ook niet willen gebruiken

12. Beïnvloedt deze test (de CRP-sneltest) uw aanvraagbeleid ten aanzien van thoraxfoto's bij patiënten die u verdenkt van een acute luchtweginfectie?

- ○ Bijna nooit
- ○ Soms
- ○ Neutraal
- ○ Vaak
- ○ Bijna altijd

13. Zou de CRP-sneltest wat u betreft de thoraxfoto als diagnosticum kunnen vervangen bij deze indicatie?

Ruimte voor eventuele opmerkingen:
