# Supplementary figures and images for: Clinical factors, C-reactive protein point of care test and chest X-ray in patients with pneumonia: A survey in primary care
Source: Eur J Gen Pract. 2019 Aug 28;25(4):229–35. doi: 10.1080/13814788.2019.1649651 (PMC6853238; doi:10.1080/13814788.2019.1649651)

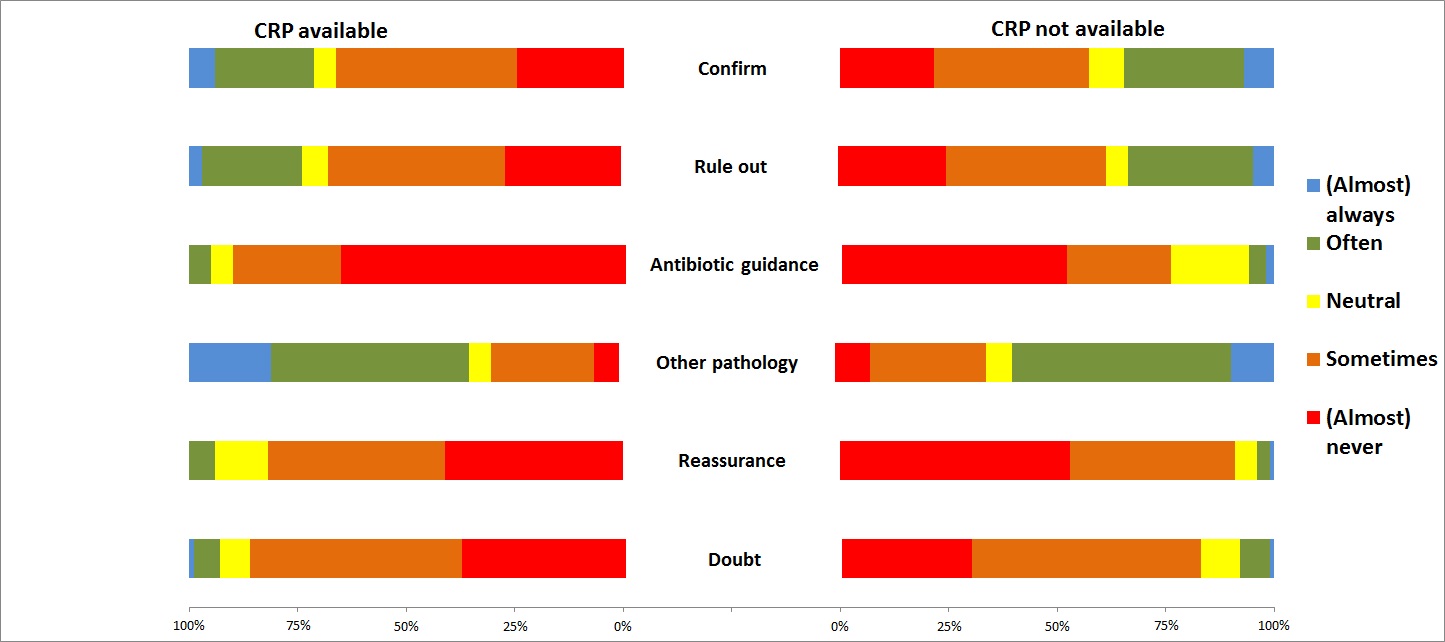

Supplement: Figure S2. Bidirectional bar chart questionnaire response from general practitioners about reasons to request a chest X ray in patients with an acute RTI [file IGEN_A_1649651_SM6387.jpg]

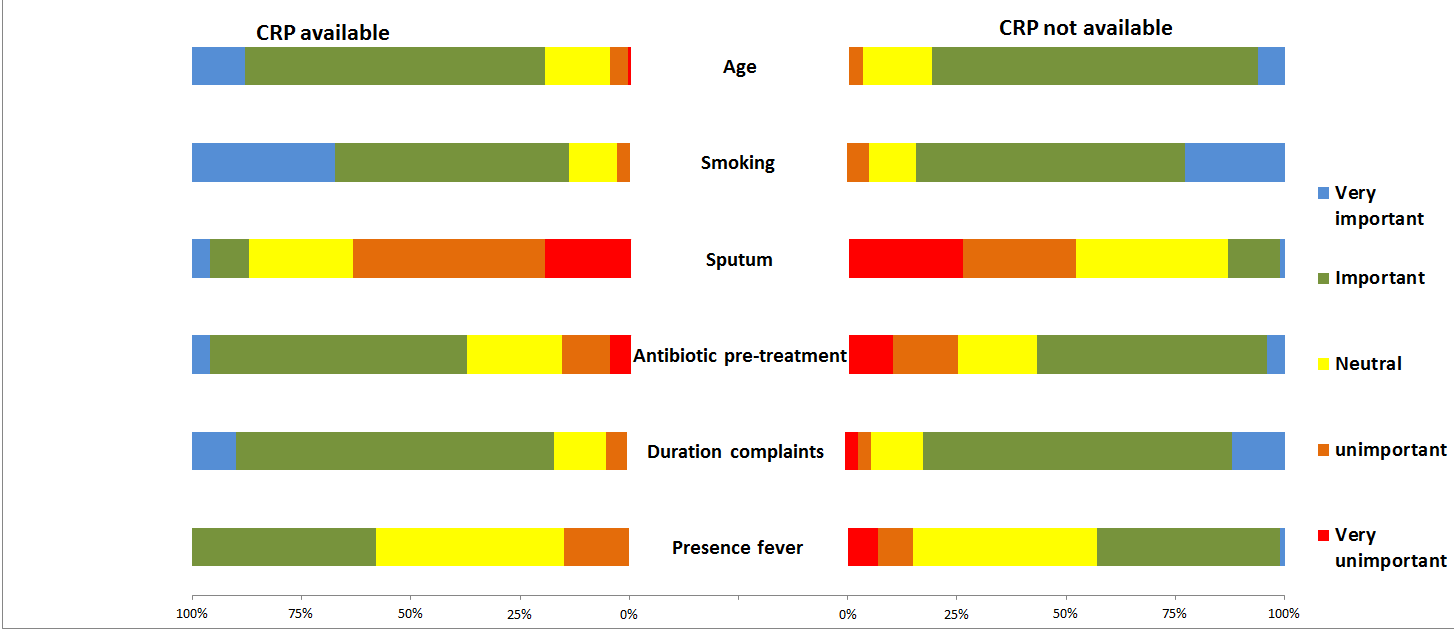

Supplement: Figure S1. Considerations to request a chest X ray with or without CRP [file IGEN_A_1649651_SM5794.png]
